# Quantitative Analysis of the Proteome and the Succinylome in the Thyroid Tissue of High-Fat Diet-Induced Hypothyroxinemia in Rats

**DOI:** 10.1155/2020/3240198

**Published:** 2020-07-23

**Authors:** Baoxiang Hu, Meng Zhao, Dandan Luo, Chunxiao Yu, Shanshan Shao, Lifang Zhao, Yashuang Yang, Xiaohan Zhang, Jiajun Zhao, Ling Gao

**Affiliations:** ^1^Department of Endocrinology, Shandong Provincial Hospital, Cheeloo College of Medicine, Shandong University, Jinan, China; ^2^Shandong Provincial Key Laboratory of Endocrinology and Lipid Metabolism, Jinan, Shandong, China; ^3^Institute of Endocrinology and Metabolism, Shandong Academy of Clinical Medicine, Jinan, Shandong, China; ^4^Department of Cardiology, Zibo Central Hospital, No. 54 Gongqingtuan West Road, Zibo 255036, Shandong, China; ^5^Department of Scientific Center, Shandong Provincial Hospital Affiliated to Shandong First Medical University, Jinan, China; ^6^Scientific Center, Shandong Provincial Hospital, Cheeloo College of Medicine, Shandong University, Jinan, China

## Abstract

Hypothyroidism is a common disease, and its molecular mechanism still needs further investigation. Lysine succinylation is found to be involved in various metabolic processes associated with hypothyroidism. We performed quantitative analysis on lysine succinylome in thyroids of rats with hypothyroxinemia, which was induced through the administration of a high-fat diet. Overall, 129 differentially expressed proteins were quantified. Downregulated proteins were enriched in the thyroid hormone synthesis and thyroid hormone signaling pathways and were mainly localized in the mitochondria. In addition, 172 lysine succinylation sites on 104 proteins were obviously changed. Decreased succinylated proteins were involved in diverse metabolic pathways and were primarily localized in mitochondria. Finally, the mitochondrial oxygen consumption rates of human normal thyroid epithelial cells were measured to further verify the role of lysine succinylation. The mitochondrial oxygen consumption rates were markedly blunted in the cells treated with palmitic acid (all *p* < 0.05), and the changes were reversed when the cells were treated with palmitic acid and desuccinylase inhibitor together (all *p* < 0.05). Thus, we theorize that the thyroid differentially expressed proteins and changed succinylation levels played potential roles in the mitochondria-mediated energy metabolism in the high-fat diet-induced hypothyroxinemia rat model.

## 1. Introduction

Thyroid hormone, synthesized and secreted by the thyroid gland, plays a crucial role in the normal development, differentiation, and metabolism of human beings [1]. Disturbances in thyroid homeostasis may result in several thyroid disorders such as hypothyroidism. Hypothyroidism is a disorder of the endocrine system that results from low production of thyroid hormone thyroxine (TT4) from the thyroid gland. This leads to metabolic dysfunction because thyroid hormone is an essential regulator of glucose-lipid metabolism and energy homeostasis. Hypothyroidism also leads to a rise in the concentration of thyrotropin (TSH) through the negative feedback of the hypothalamus-pituitary-thyroid axis [2]. Primary hypothyroidism, caused by a dysfunction of the thyroid itself, is the main cause of hypothyroidism [3]. The onset of hypothyroidism in adults is often subtle presenting with a range of nonspecific symptoms. However, severe untreated hypothyroidism may result in poor prognoses, such as heart failure, psychosis, and even coma [3]. So far, possible measures for the treatment of hypothyroidism include improvements in symptoms and prevention of adverse event. Undoubtedly, hypothyroidism places a huge burden on the economy and greatly lowers the quality of the patient's life. Thus, it is essential to investigate the pathogenesis and explore novel treatment strategies.

Posttranslational modifications (PTMs), which refer to covalent modifications introduced to amino acids of proteins either enzymatically or nonenzymatically, are key mechanisms for increasing proteomic diversity and exert crucial effects on biological function in a variety of species [4–7]. PTMs modulate protein properties through proteolytic cleavage of regulatory subunits, addition of a modified group to one or more amino acids, or degradation of entire proteins, thus determining activity status, localization, turnover, and interactions with other molecules [8].

Lysine, as the most common posttranslation modified amino acid residue, is critical for the formation of protein structures and regulation of protein functions. Lysine residues can be subjected to various PTMs, such as methylation, acetylation, biotinylation, ubiquitination, ubiquitin-like modifications, propionylation, and butyrylation [9–13]. These lysine PTMs play important roles in cellular physiology and pathology, thereby influencing almost all aspects of cell biology and pathogenesis [14–17]. Lysine succinylation is one of significant posttranslational protein modifications, which can occur on cytosolic, nuclear, and mitochondrial proteins by a nonenzymatic chemical reaction [18] and enzymatic catalytic reaction. The former succinylation originates directly from succinyl-CoA, which can be generated from the TCA cycle, lipids, and amino acid metabolism, and the enzymatic succinylation of lysine takes place by lysine succinyltransferase. Lysine succinylation has been identified and verified as an important form of PTM and is involved in a diverse array of cellular functions associated with thyroid diseases [19–21]. Cinzia Puppina et al. have found that acetylated levels of lysine at positions 9–14 of H3 histone (H3K9-K14ac) were significantly higher in follicular adenomas, papillary thyroid carcinomas, follicular thyroid carcinomas, and undifferentiated carcinomas than in normal tissues [22]. Andrea Henze et al. reported that oxidative modifications of Cys10 seemed to affect the binding of T3 to transthyretin and provided a sensitive mechanism for adjusting thyroid hormone availability [23]. The role of lysine succinylation in thyroid diseases is still unknown and needs further investigation.

Our previous studies [24, 25] have found that excess intake of dietary fat induced decreased serum TT_4_ and FT_4_ concentrations in parallel with elevated serum TSH concentration, as well as abnormal morphology and lipid profile change of the thyroid gland, providing evidence for the correlation between lipid profiles and organ function, as well as a new prospect for understanding the pathogenesis of hypothyroidism. However, the underlying molecular mechanism remains unclear and needs further investigation.

Liquid chromatography-tandem mass spectrometry- (LC-MS/MS-) based proteomic analysis has emerged as a powerful tool for studying disease mechanisms due to its high throughput and accuracy [20, 26, 27]. In the present study, to investigate which PTM played a role in hypothyroxinemia, we detected four types of lysine acylations by western blotting, including succinylation, crotonylation, 2-hydroxybutyrylaion, and malonylation. A significant change in lysine succinylation was observed in the HFD group relative to the control group. Then, we carried out label-free-based quantitative analyses on the global proteome and lysine succinylome of thyroid tissues in the HFD-induced thyroid dysfunction rat model using LC-MS/MS methods. A series of bioinformatics analyses were conducted to explore the underlying molecular mechanisms of hypothyroxinemia and lysine succinylation's involvement. We aimed to explore the association between lysine succinylation and hypothyroidism and to evaluate potential diagnostic biomarkers and therapeutic targets.

## 2. Materials and Methods

### 2.1. Experimental Design and Workflow

We compared the protein expression profile and succinylation level in the rat thyroid tissue between a high-fat diet (HFD) study group and a chow-diet (CD) control group. The experiment procedures consisted of four key steps as follows: (1) the establishment and sample collection in a hypothyroxinemia rat model, as previously described [24, 25]; (2) label-free-based quantitative proteomics, including protein extraction, trypsin digestion, high-performance liquid chromatography (HPLC) fractionation, and antibody-based affinity enrichment of lysine succinylated peptides; (3) LC-MS/MS analyses; and (4) bioinformatics analyses. Three biological replicates were performed for the global proteome and succinylome analyses. The procedure is described in detail in the following paragraphs.

### 2.2. Animal Model and Ethics Statement

Twenty-six male SD rats at 6-week-old (Beijing Vital River Laboratory Animal Technology Co., Ltd., Beijing, China) weighing 190–210 g were fed in the experimental animal center of Shandong Provincial Hospital, Shandong University. The rats were maintained at a constant temperature and humidity and were rendered a 12 h : 12 h light–darkness cycle. The rats were randomly and equally divided into the CD control group (*n* = 13) and the HFD study group (*n* = 13) (the detailed composition of fatty acids in diets is shown in Supplementary Table S1). The animals were weighed weekly and fed for 24 weeks. At the 24^th^ week of feeding, all rats were fasted for 12 hours before sacrifice. All experiment protocols were approved by the Animal Ethics Committee of Shandong Provincial Hospital, Shandong University.

### 2.3. Serum Thyroid Function Parameters Analysis

Serum TT_4_, FT_4_, and TSH concentrations were measured at the end of the experiment. Blood samples were collected by inferior vena cava puncture. Serum TT_4_, FT_4_, and TSH were measured by using ELISA kits (CUSABIO, Wuhan, China). All procedures were carried out in accordance with the instructions provided by the manufacturers.

### 2.4. Protein Extraction and Trypsin Digestion

Thyroid tissue samples from 4, 4, and 5 rats were pooled as three biological replicates, respectively. Each sample was grinded by liquid nitrogen into cell powder and then transferred to a 5 mL centrifuge tube. Following the addition of four volumes of lysis buffer (8 M urea, 1% protease inhibitor cocktail, 3 *μ*M TSA, 50 mM NAM, and 2 mM EDTA) into the centrifuge tube, sonication on ice was performed three times. After centrifugation at 12,000 g at 4°C for 10 min, the remaining debris was removed, and the supernatant was collected. Finally, a BCA kit (Beyotime Institute of Biotechnology, Shanghai, China) was utilized to determine the protein concentration according to the manufacturer's instructions.

In preparation for digestion, the protein solution was reduced (5 mM dithiothreitol, 30 min, 56°C) and alkylated (11 mM iodoacetamide, 15 min, room temperature in darkness). The urea concentration was then diluted to less than 2 M by adding 100 mM NH_4_HCO_3_. Finally, trypsin (Promega Corporation, Fitchburg, Wisconsin, United States) was added at 1 : 50 enzyme-to-substrate mass ratio and incubated overnight for the first digestion, followed by the addition of trypsin at 1 : 100 enzyme-to-substrate mass ratio for an additional 4 h digestion.

### 2.5. HPLC Fractionation and Antibody-Based Affinity Enrichment

The tryptic peptides were then fractionated into several fractions by high-pH reverse-phase HPLC using Agilent 300 Extend C18 columns (5 *μ*m particles, 4.6 mm ID, 250 mm length). Briefly speaking, peptides were first separated into 60 fractions with a gradient of 8%–32% acetonitrile (ACN, pH 9.0) for over 60 min. Afterwards, the peptide fractions were combined into 4 fractions and dried by vacuum centrifuging.

Enrichment was implemented by immunoprecipitation in accordance with previous studies [28, 29]. Briefly, to enrich lysine-succinylation modified peptides, tryptic peptides were dissolved in NETN buffer (100 mM NaCl, 1 mM EDTA, 50 mM Tris-HCl, 0.5% NP-40, and pH 8.0) and then incubated overnight with prewashed antisuccinyl lysine antibody agarose beads (catlog no. PTM402; PTM Bio, Hangzhou, China) at 4°C with gentle shaking. Finally, the bound peptides were eluted from the beads with 0.1% trifluoroacetic acid (TFA), combined, and vacuum-dried. Before LC-MS/MS analysis, the obtained peptides were desalted with C18 ZipTips (Millipore) according to the manufacturer's instructions.

### 2.6. LC-MS/MS Detection, Database Search, and Quantification Analysis

The tryptic peptides were resuspended in solvent A (0.1% formic acid in 2% ACN) and then directly loaded onto a reversed-phase analytical column (15 cm length, 75 *μ*m ID; PTM bio, Hangzhou, China). A constant flow rate of 700 nl/min was established on an EASY-nLC 1000 UPLC system, with a gradient consisting of 9%–25% solvent B (0.1% formic acid in 90% ACN) for 38 min, 25%–40% for 14 min, climbing to 80% for 4 min, and holding at 80% for the last 4 min.

The peptides were subjected to a nanospray ionization (NSI) source, followed by tandem mass spectrometry (MS/MS) in a Q Exactive^TM^ Plus (Thermo Fisher Scientific) coupled online to an ultraperformance liquid chromatograph (UPLC). Intact peptides and succinylated peptides were detected in the Orbitrap at a resolution of 70,000 and a m/z scan ranging from 350 to 1800. The peptides were then selected using 28% normalized collision energy (NCE) for MS/MS analyses, and the ion fragments were detected using the Orbitrap at a resolution of 17,500. Data-dependent acquisition (DDA) procedures that alternated between one MS scan followed by 15 and 20 MS/MS scans were applied to collect the top 15 and 20 precursor ions of peptides and succinylated peptides above a threshold ion count of 10,000 in the MS survey scan with 30.0 s and 15.0 s dynamic exclusions, respectively. The electrospray voltage applied was 2.1 kV. The automatic gain control (AGC) was utilized to prevent overfilling of the ion trap, and 50,000 ions were accumulated for the generation of MS/MS spectra. The maximum injection time was set as 200 ms and 100 ms for peptides and succinylated peptides, respectively.

The acquired MS/MS data were processed and analyzed with the MaxQuant search engine (v.1.5.2.8). Tandem mass spectra were searched against the UniProt rat database (29,795 sequences) concatenated with protein sequences of common contaminants (such as hemoglobin, keratin, and lactoglobulin) and a reverse decoy database. Trypsin/P was specified as a cleavage enzyme allowing up to two missing cleavages, as well as five modifications per peptide. The mass error was set as 20 ppm in the first search and 5 ppm in the main search for precursor ions and 0.02 Da for fragment ions. Carbamidomethyl on cysteine was specified as a fixed modification, whereas acetylation on the protein *N*-terminal and oxidation on methionine were specified as variable modifications. For succinylome analysis, succinylation on lysine was also set as variable modifications. The false discovery rate (FDR) thresholds for the identification of PTM levels, peptides, and proteins were adjusted to 0.01. The minimum length of peptide was set as 7 amino acid residues.

The quantitative values of each sample in three replicates were obtained by LFQ intensity. The first step is to calculate the differential concentration of the protein between the two samples. First, calculate the average value of the quantitative values of each sample in multiple replicates, and then, calculate the ratio of the average values between the two samples. The ratio is used as the final quantitation. For normalization to succinylated peptides, the naked intensities of succinylated peptides were first measured and then were divided by the corresponding protein intensities [30]. To calculate the significant *p* value of differential concentration between two samples, the relative quantitative values of each sample were taken as log2 transform (so that the data conform to the normal distribution), and *p* value was calculated by the two-sample two-tailed *t*-test method. *p* value < 0.05 and protein ratio >1.5 were regarded as upregulation. *p* value < 0.05 and protein ratio <1/1.5 were regarded as downregulation.

### 2.7. Bioinformatics Analysis

The raw proteome and succinylome mass spectrometric data have been deposited to the ProteomeXchange (https://www.ebi.ac.uk/pride) with identifier PXD012814. Gene ontology (GO) analyses were derived from the UniProt-GOA database (http://www.ebi.ac.uk/GOA/) and GO annotation (http://geneontology.org/) to classify all identified proteins into three categories: biological process, cellular component, and molecular function. A cutoff of absolute fold change ≥1.5 was employed to identify the differentially expressed proteins. The functional pathways of all quantified proteins or succinylated proteins were annotated by performing the Kyoto Encyclopedia of Genes and Genomes (KEGG) analysis (http://www.genome.jp/kegg/). The functional enrichment analyses were carried out to reveal the differentially expressed proteins enriched in all identified proteins and succinylated proteins. When performing bioinformatics analysis, a two-tailed Fisher's exact test was performed, and a corrected *p* value < 0.05 was considered significant. Protein-protein interactions were analyzed by STRING (http://string-db.org/) using differential proteins and succinylated proteins with significant abundance changes as input. The required confidence score was set as >0.700 for highly confident interactions. The results were visualized using the Cytoscape package.

### 2.8. Cell Culture and Reagents

The human normal thyroid epithelial cell line Nthy-ori3-1 (ECACC, Wiltshire, UK) was cultured in RPMI-1640 (Gibco, Thermo Fisher Scientific, Inc., Waltham, MA, USA) supplemented with 10% fetal bovine serum (FBS; ExCell Bio, Shanghai, China), penicillin (100 IU/ml), streptomycin (100 IU/ml), and L-glutamine (2 mM) at 37°C in a humidified atmosphere containing 5% CO_2_.

For the measurement of mitochondrial functions, briefly, palmitic acid (PA) from 50 mM stock solution was warmed and freshly diluted in 2.5 mM BSA-PBS. The diluted PA solution was warmed to clear in a 55°C water bath. Then, the solution of PA and nicotinamide (NAM) was added to the cultures, respectively. Cells were divided into three groups according to different treatments (the NC group, the PA group, and the PA + NAM group).

For immunoprecipitation, cells divided into four groups (the control group, the NAM group, the PA group, and the NAM + PA group). NAM, PA, and NAM + PA groups were treated with NAM, PA, and both NAM and PA, respectively. All reagents were purchased from Sigma-Aldrich (Saint Louis, USA) unless otherwise stated.

### 2.9. Measurement of Mitochondrial OCR

The measurement of OCR was performed using an XF96 Analyzer (Seahorse Bioscience, USA) according to the manufacturer's instructions. In brief, approximately 7 × 10^3^ cells per well were seeded onto the Seahorse XF96 cell culture microplate (Seahorse Bioscience, USA) and cultured for 24 hours. After the administration of PA (0.2 mM) and NAM (10 mM), the cells were cultured for another 24 hours. Then, the microplate was incubated in the low-buffered and nonbicarbonated assay medium (XF base medium with 2 mM glutamine, 1 mM sodium pyruvate, and 25 mM glucose) in a non-CO_2_ incubator at 37°C for 1 hours. Then, OCRs were measured in an XFe 96 extracellular flux analyzer (Seahorse Bioscience) for 3 periods with 3 min of mixing in each cycle. The results were normalized to the corresponding total protein concentration per well.

### 2.10. Immunoprecipitation

Cells were cultured for 24 hours. After the administration of PA (0.2 mM) and NAM (10 mM), the cells were cultured for another 24 hours. Then, cells were washed three times with ice-cold PBS and lysed in 1 ml ice-cold RIPA lysis buffer (50 mM Tris, 150 nM NaCl, 1% sodium deoxycholate, 1% Triton-X-100, 1 mM EDTA, 0.1% SDS, 10 mM NaF, 1 mM sodium orthovanadate, 1 mM PMSF, and 10 mM NAM). Cells were scraped off from plates, and cell lysates were centrifuged at 12,000 g for 15 minutes. Supernatants were collected, and protein concentration was measured by a BCA kit. 500 *μ*g of total protein was used for IP. Proteins were incubated with the primary antibody overnight at 4°C with gentle rocking. Immunocomplexes were immunoprecipitated using protein A-agarose beads. The immunoprecipitate was washed four times with lysis buffer. Finally, each bead pellet was resuspended in 20 *μ*l of 2× reducing loading buffer (130 mM Tris pH 6.8, 4% SDS, 0.02% bromophenol blue, 20% glycerol, and 100 mM DTT) and boiled at 100°C for 5 min. Samples were stored at −80°C, followed by Western blotting.

### 2.11. Western Blotting

Equal amounts of protein from different samples were subjected to 8% SDS-PAGE, followed by electrotransfer from the gel to polyvinylidene difluoride membranes (Millipore).The membrane was blocked with 5% (w/v) skim milk in TBST and incubated overnight at 4°C with the pan antisuccinyl lysine antibody (1 : 1000 dilution; PTM Biolabs), anti-sirt5 antibody (1 : 1000 dilution; CST), and anti-GAPDH antibody (1 : 5000; Proteintech, 66009-1-l g). Following the primary antibodies, the membranes were incubated with the corresponding secondary antibodies at 1 : 5000 dilution for 1 h at room temperature. Immune complexes were detected using an Amersham Imager 600 (General Electric Company). The same membrane was reincubated with anti-GAPDH antibodies. The GAPDH protein was used as a loading control for total proteins.

### 2.12. Statistical Methods

Quantitative data were presented as the mean ± SEM and were processed using GraphPad Prism 6.0 (La Jolla, CA, USA) and SPSS version 22.0 (Chicago, IL, USA). One-way ANOVA followed by Turkey's post hoc test was performed for multiple comparisons. A *p* value < 0.05 was considered significant when comparing HFD thyroid samples with their corresponding CD thyroid samples.

## 3. Results

### 3.1. HFD-Induced Hypothyroxinemia

**Figure 1:**
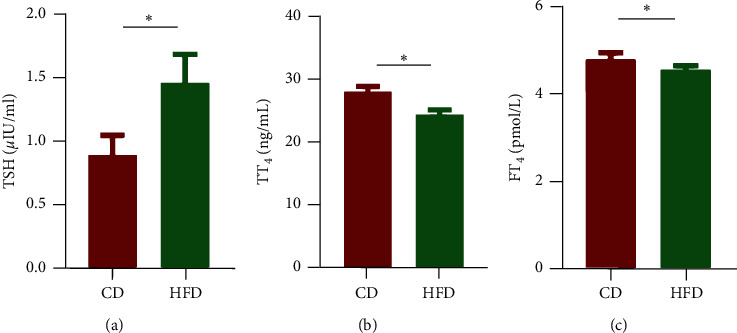
Thyroid hormones measurement. Serum TSH (a), TT_4_ (b), and FT_4_ (c) were measured using ELISA kits in rats in the chow-diet (CD) control group or the high-fat diet (HFD) study group. Hypothyroxinemia in the HFD group was observed. Error bars represent the mean ± SD. ^*∗*^*p* < 0.01 versus the CD group.

To observe the thyroid function, we measured serum TT_4_, FT_4_, and TSH. As shown in Figure 1, the HFD group exhibited decreased concentration of TT_4_ (*p* < 0.01) and FT_4_ (*p* < 0.01) in parallel with elevated concentration of TSH (*p* < 0.01). These results indicate that the establishment of the hypothyroxinemia rat model was successful.

### 3.2. General Characterization of the Quantitative Proteome in Rat Thyroid Tissues

**Figure 2:**
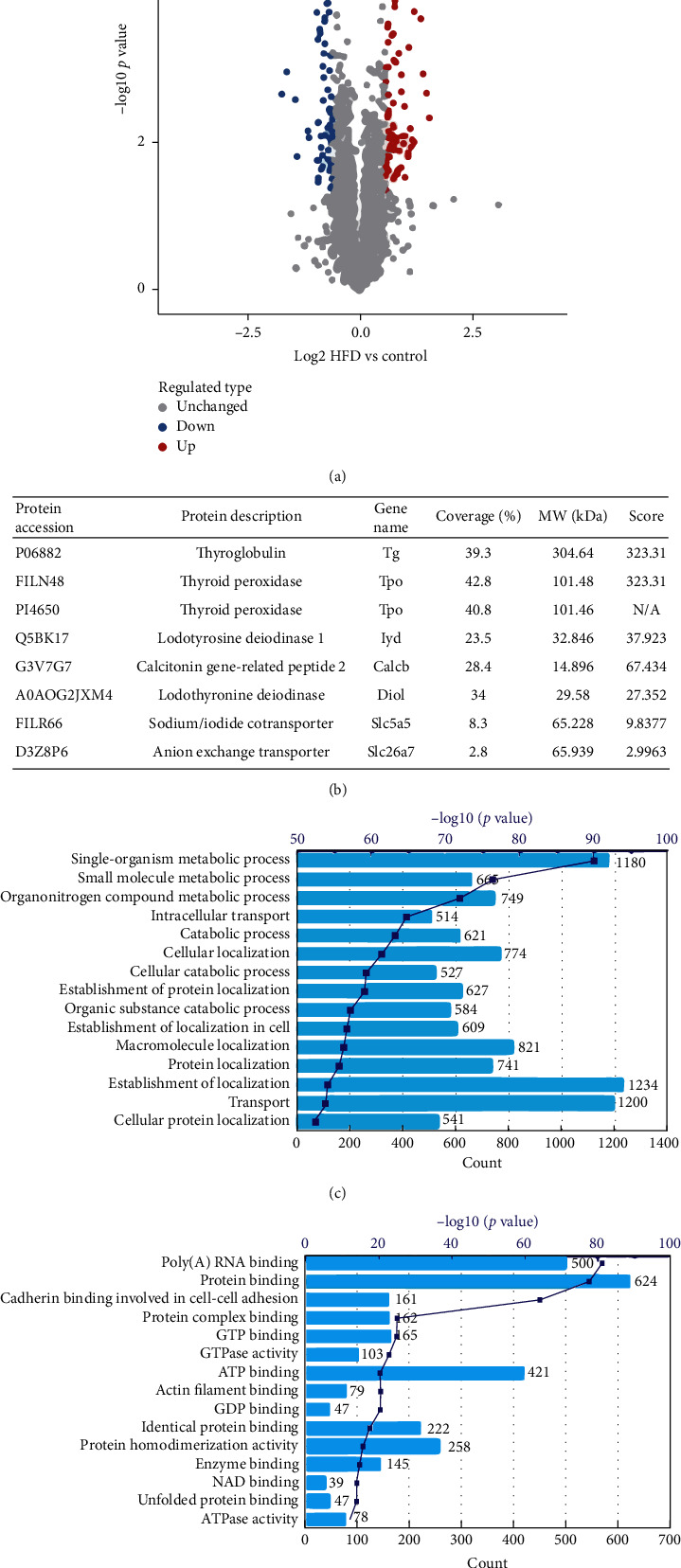
General characterization of the quantitative proteome. (a) Volcano plot illustrating significantly differential abundant proteins in proteome analysis. The −log10 (*p* value) is plotted against the log2 (ratio HFD/control). (b) Table illustrating the specific proteins in thyroid detected. (c, d) GO biologic process and molecular function enrichment analysis, respectively; bars length represents genes number and dots indicate −log10 (*p* value) corresponding GO items.

Label-free-based quantitative proteomics was performed using HPLC fractionation and high-resolution LC-MS/MS analysis. Pairwise Pearson's correlation coefficients displayed sufficient reproducibility of the experiment (Supplementary Figure S1A in the Supplementary Material for comprehensive image analysis). A total of 3869 proteins were identified, among which 2982 proteins were quantitative (Supplementary Table S2). Differentially expressed proteins were filtered with a fold-change threshold >1.5 (*p* value <0.05) for upregulation and a ratio < 1/1.5 (*p* value <0.05) for downregulation in the thyroid of the rats with HFD relative to the control. A total of 129 proteins were quantified as differentially expressed proteins between the two groups, including 69 upregulated and 60 downregulated proteins, which is exhibited by volcano plot (Figure 2(a)). Then, these differentially expressed proteins were annotated by performing intensive bioinformatics analyses.

Comparing rat thyroid proteome data with Protein Atlas Publica database, some hallmark proteins in the thyroid tissue can be found in our detected results (Figure 2(b)). For example, thyroglobulin (Tg) is the most abundant among all proteins in MS detected, which acts as a substrate for the synthesis of the thyroid hormones, thyroxine (T4) and triiodothyronine (T3). Thyroid peroxidase is also high level expressed, which is involved in the pathway of thyroid hormone biosynthesis. Other specific proteins such as iodotyrosine deiodinase 1 and calcitonin gene-related peptide 2 in Protein Atlas thyroid database exist in our protein detected table too (Supplementary Table S2). All of these proved our mass spectrum proteomics qualification, and quantification is credible.

GO biological process and molecular function enrichment analysis were performed to all quantified proteins. GO biologic process enrichment analysis shows that the most significant enrichment is metabolic process, including single-organism metabolic process, small molecule metabolic process, and organonitrogen compound metabolic process (Figure 2(c)). For GO molecular function enrichment, poly(A) RNA binding, protein binding, and cadherin binding involved in cell-cell adhesion are top three GO items.

### 3.3. Enrichment Analysis of the Differentially Expressed Proteins

Enrichment analyses were performed to identify GO terms, KEGG pathways, and domains that were significantly enriched.

**Figure 3:**
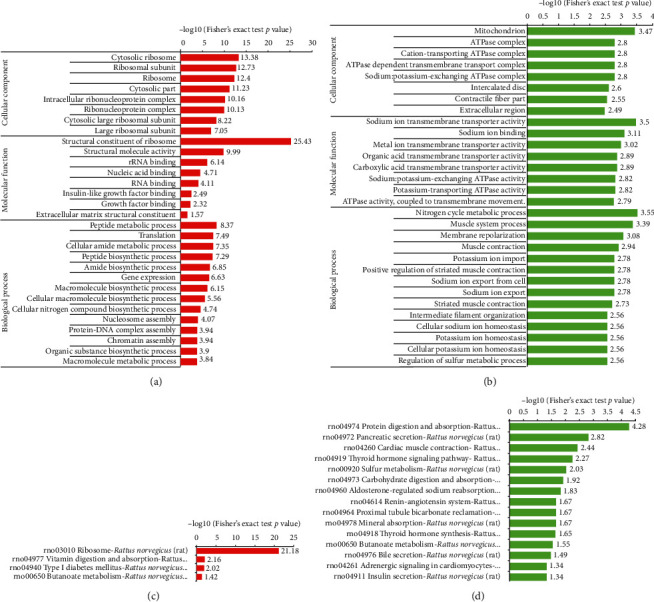
Enrichment analyses of the differentially expressed proteins: (a) upregulated and (b) downregulated proteins were examined by the GO functional enrichment; (c) upregulated and (d) downregulated proteins were examined by the KEGG pathway analysis. Upregulated and downregulated proteins were defined as >1.5 folds and <1/1.5 compared to the control, respectively.

GO analyses were conducted to characterize the biological processes and molecular functions of the differentially expressed proteins. As shown in Figures 3(a) and 3(b), among cellular components, the expression of proteins localized to the ribosome and ribosome subunit increased, while the expression of proteins localized to the mitochondrion and ATPase complex significantly decreased. Among the molecular functions, the structural constituents of ribosomes and the structural molecule activity were upregulated, while the ATPase activity was downregulated. In the biological process category, the upregulated proteins were markedly enriched in several metabolic processes (including peptide, cellular amide, and macromolecule metabolism), biosynthetic processes (such as peptide, amide, macromolecule, and organic substance biosynthesis), and translation. In contrast, some downregulated proteins were enriched in a number of metabolic processes including the nitrogen cycle and sulfur metabolism.

The KEGG pathway enrichment analyses were also performed to further investigate the functions of these differentially expressed proteins. Consistent with the results of GO analyses, the results show that the ribosome pathway was the most prominent enriched pathway for upregulated proteins (Figure 3(c)). Meanwhile, downregulated proteins were observed to be enriched in the thyroid hormone signaling pathway and thyroid hormone synthesis, indicating that these pathways may play essential roles in the development of hypothyroxinemia (Figure 3(d)).

### 3.4. General Characterization of Quantitative Succinylome in Rat Thyroid Tissues

**Figure 4:**
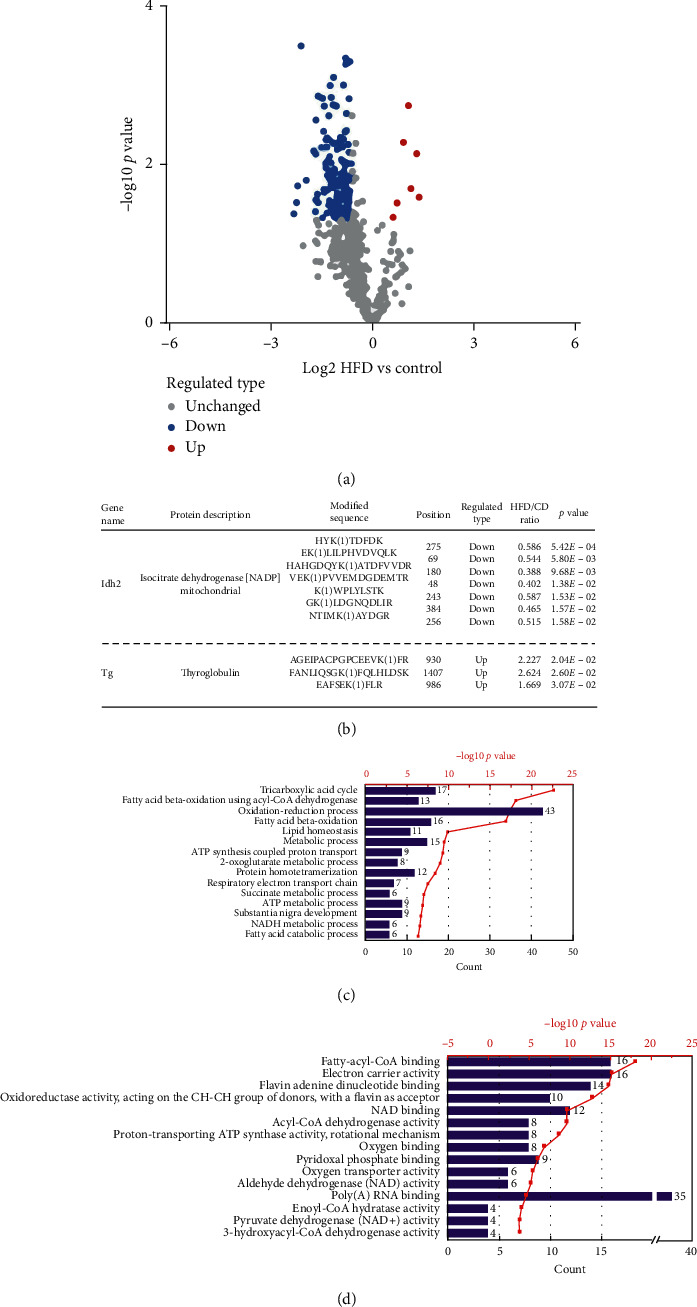
General characterization of the quantitative succinylated proteins. (a) Volcano plot illustrating significantly differential abundant succinylation sites analysis. The −log10 (*p* value) is plotted against the log2 (ratio HFD/control). The −log10 (*p* value) is plotted against the log2 (ratio HFD/control). (b) Table illustrating significant differential upregulated or downregulated succinylation site corresponding peptides. (c, d) GO biologic process and molecular function enrichment analysis, respectively; bars length represents genes number and dots indicate −log10 (*p* value) corresponding GO items.

Label-free-based quantitative lysine succinylome analysis was performed using antibody-based affinity enrichment, followed by LC-MS/MS analysis. Altogether, 685 succinylation sites in 250 proteins were identified, among which 621 succinylation sites on 229 proteins were quantified and normalized to the proteome data (Supplementary Table S3). With a quantification ratio of >1.5 (*p* value < 0.05) as the upregulation threshold and <1/1.5 (*p* value < 0.05) as the downregulation threshold, 172 succinylation sites corresponding to 104 proteins showed different succinylation levels in three repeated experiments (7 upregulated succinylated sites on 5 proteins, 165 downregulated succinylated sites on 99 proteins, and the HFD group compared with the CD group), which is exhibited by volcano plot (Figure 4(a)). The average peptides mass error was <10 ppm, indicating a high mass accuracy of the MS data (Supplementary Figure S1B in the Supplementary Material for comprehensive image analysis). The lengths of the most identified peptides were 8–20 amino acid residues (Supplementary Figure S1C in the Supplementary Material for comprehensive image analysis).

Compared with the CD group, most lysine succinylation on different proteins undergo downregulated change in the HFD group, moreover, these succinylated proteins are located in mitochondria, including ATP synthase complex and isocitrate dehydrogenase (IDH2). However, several lysine sites succinylation are upregulated on thyroglobulin (Figure 4(b)). GO biologic process enrichment analysis reveals that the tricarboxylic acid cycle, fatty acid beta-oxidation using acyl-CoA dehydrogenase, oxidation-reduction process, fatty acid beta-oxidation, and lipid homeostasis are top five significant GO items (Figure 4(c)). And, molecular function enrichment analysis (Figure 4(d)) shows that fatty-acyl-CoA binding and related metabolism are enriched significantly.

### 3.5. Enrichment Analysis of the Differentially Changed Succinylated Proteins

**Figure 5:**
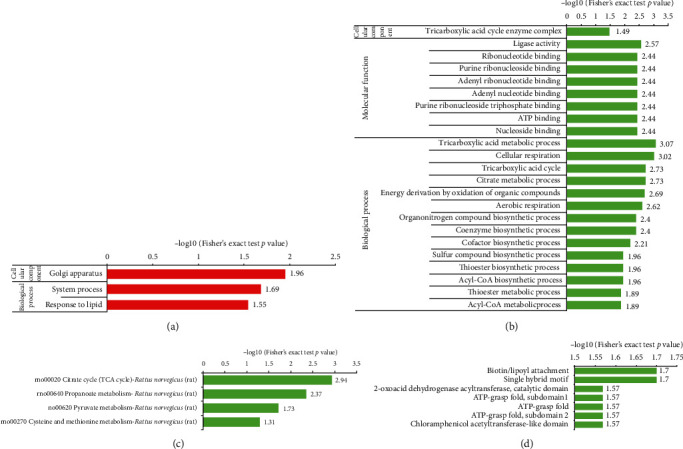
Enrichment analyses of the differentially changed succinylated proteins: (a) upregulated and (b) downregulated succinylated proteins were classified by the GO functional enrichment; downregulated succinylated proteins were examined by (c) KEGG pathway analysis and (d) domain enrichment analysis.

As shown in Figures 5(a) and 5(b), among cellular components, the downregulated succinylated proteins mainly exhibited the tricarboxylic acid cycle (TCA cycle) enzyme complex. The most enriched molecular function was the ligase activity. In the biological process category, proteins related to the system process that responded to the lipids were enriched among the upregulated succinylated proteins, while proteins related to the TCA metabolic process, cellular respiration, TCA cycle, citrate metabolic process, energy derivation by oxidation of organic compounds, and aerobic respiration were enriched among the downregulated succinylated proteins.

The KEGG pathway enrichment analysis indicated that the citrate cycle (TCA cycle) was the most enriched pathway among the downregulated succinylated proteins. In addition, propanoate metabolism and pyruvate metabolism related pathways were also enriched (Figure 5(c)). Protein domain analysis revealed that the top two significantly enriched terms were biotin/lipoyl attachment and single hybrid motif (Figure 5(d)).

### 3.6. Differential Proteins and Succinylated Proteins PPI Analysis

To better understand the function of lysine succinylation and hypothyroidism pathology, the differential proteins and succinylated proteins were subjected to a protein-protein interaction (PPI) network analysis using the STRING database (Vision 11.0). STRING defines a metric called “confidence score” to define interaction confidence; we fetched all interactions that had a confidence score ≥0.7 (high confidence). A network of protein-protein interactions was generated and clustered with the Markov cluster (MCL) algorithm [31], which was then visualized using the Cytoscape program (Vision 3.7).

**Figure 6:**
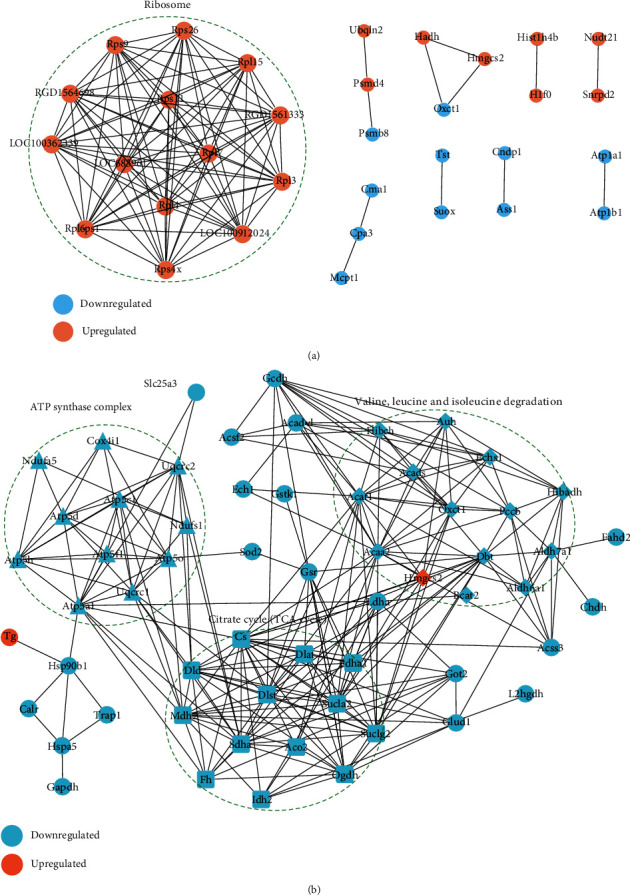
Differential proteins and succinylated proteins interacting net analysis. (a) Differential proteins PPI net. (b) Differential succinylated proteins PPI net.

Differential protein-protein net analysis reveals that ribosome proteins interaction is highly clustered, and the proteins of this PPI net cluster is characterized with upregulated coexpression (Figure 6(a)). The differential succinylated proteins interacting net (Figure 6(b)) shows three function clusters: the citrate cycle (TCA cycle), ATP synthase complex, and valine, leucine, and isoleucine degradation, and all of them are downregulated succinylation.

### 3.7. Measurement of Mitochondrial OCR

**Figure 7:**
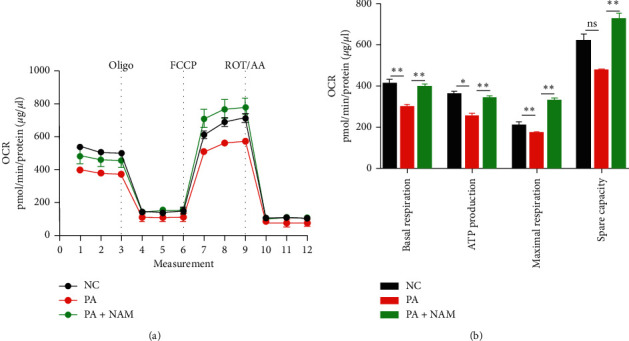
Human normal thyroid epithelial cells were isolated and cultured. The cells were treated with and without palmitic acid (PA) in the presence and absence of a desuccinylation inhibitor (NAM) for 24 hours, respectively. Mitochondrial OCRs were measured using the Seahorse XF96 analyzer (mean ± SEM, *n* = 7–8). OCRs related to the mitochondrial basal respiration, ATP production, maximal respiration, and spare capacity were analyzed and normalized to the corresponding total protein concentration per well, respectively, (ns *p* > 0.05, ^*∗*^*p* < 0.05, ^*∗∗*^*p* < 0.01). Each datum was obtained from independent three days. One-way ANOVA followed by Turkey's post hoc test was performed for multiple comparisons.

To further investigate the role of fatty acids in mitochondrial function and to avoid cofounding factors *in vivo*, OCR representing levels of mitochondrial function were measured in normal human thyroid epithelial cells. As shown in Figure 7, mitochondrial OCRs related to basal respiration, ATP production, and maximal respiration are markedly blunted by palmitic acid exposure (all *p* < 0.05), and the changes were reversed when the cells were treated with palmitic acid and desuccinylase inhibitor together (all *p* < 0.05).

### 3.8. Verification of Protein Succinylation Levels

**Figure 8:**
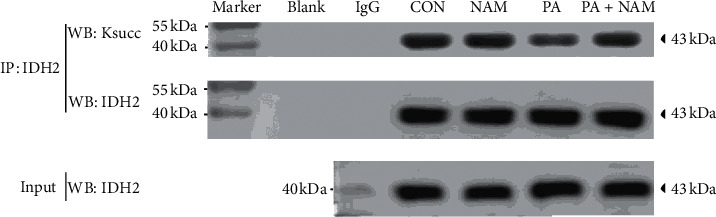
Human normal thyroid epithelial cells were isolated and cultured. The cells were treated with and without palmitic acid (PA) in the presence and absence of a desuccinylation inhibitor (NAM) for 24 hours, respectively. Total proteins of each group were subjected to immunoprecipitation (IP) with an antibody of isocitrate dehydrogenase 2 (IDH2). Succinylation status and expression of IDH2 were evaluated by Western blotting analysis. IDH2 expression showed no obvious change. IDH2 succinylation was inhibited by PA treatment and showed no obvious change in response to NAM alone. After treatment of both PA and NAM, IDH2 succinylation level was recovered compared with PA treatment.

To determine the variation of protein succinylation in cell line, we performed protein immunoprecipitation assays coupled with Western blotting to detect isocitrate dehydrogenase 2 (IDH2) succinylation levels. IDH2 was known as a critical enzyme in the tricarboxylic acid cycle. Nthy-ori3-1 cells were treated with NAM or PA or both NAM and PA, respectively. The result showed that IDH2 was indeed succinylated, and its succinylation was inhibited by PA treatment. Although IDH2 succinylation did not show obvious change in response to NAM, both PA and NAM treatment made IDH2 succinylation level recovered compared with PA treatment (Figure 8).

## 4. Discussion

In present study, a HFD-induced hypothyroxinemia rat model was constructed according to our previous studies [24, 25]. Excess intake of dietary fat induced significant thyroid dysfunction and hypothyroxinemia in rats by decreasing the expression of thyroid hormone synthesis-related proteins, providing evidence for the correlation between lipid profiles and organ function. Then, we employed a quantitative proteomics strategy and LC-MS/MS-based enrichment to investigate global protein and succinylation profiles in thyroid tissues. We identified 129 differentially expressed proteins and 172 differentially expressed succinylation sites, among which several proteins and succinylation sites were localized in the mitochondria and associated with mitochondrial function. To confirm the alterations in mitochondrial respiratory activities, OCR was further employed to verify the LC-MS/MS results.

The proteome data demonstrated changes in the metabolic processes in the HFD-induced hypothyroxinemia rat model. Benard et al. presented a proteome-wide study in HFD-fed mice and detected that fifty-four percent of those differentially expressed proteins were involved in metabolic processes [32]. In addition, Yang et al. revealed that the metabolic pathways of differentially expressed proteins were possibly related to the HFD-induced decline in male rats' fertility [33]. Furthermore, Tu et al. found that the protein expression levels of intracranial and extracranial atherosclerosis in HFD-fed rabbits were different, which facilitated the diagnosis and treatment of cerebral arteriosclerosis [34]. These proteomics studies provided molecular understandings of HFD-induced pathology and identified potential targets for the development of therapeutics for metabolic syndromes. Thus, we theorize that HFD may play an important role in the formation of hypothyroidism [35, 36].

Recent studies have focused on protein PTMs to identify the potential mechanisms of several diseases. Various types of PTMs such as phosphorylation, lysine acetylation, ubiquitination, propionylation, crotonylation, and succinylation have been discovered with the development of mass spectrometry technology [7, 12, 37]. Particularly, the role of PTMs in regulating cellular energy metabolism has been demonstrated. To investigate which PTM played a role in hypothyroxinemia, we detected four types of lysine acylations by western blotting, including succinylation, crotonylation, 2-hydroxybutyrylaion, and malonylation. A significant change in lysine succinylation was observed in the HFD group relative to the control group. Lysine succinylation, as a newly identified PTM in proteins, is widespread in diverse organisms and impacts various metabolic pathways [38–41]. Thus, we investigated the quantitative protein succinylome in the HFD-induced hypothyroxinemia rat model, with the goal of exploring the possible role of lysine succinylation in HFD-induced hypothyroxinemia.

At the succinylome level, our data indicated a close relationship between lysine succinylation and mitochondria-mediated metabolic regulation. As we all know, mitochondria participate in the metabolism of amino acids, lipids, cholesterol, steroids, and nucleotides. Perhaps, most importantly, mitochondria play a fundamental role in cellular energy metabolism (including the fatty acid *β*-oxidation and the respiratory chain), which is essential to diverse cellular functions and developmental processes [35, 36]. Recent protein succinylome studies in human renal cell carcinoma tissues have shown that the glycolysis pathway might be regulated through lysine succinylation and play a potential role in renal cell carcinoma progression [42]. Additionally, Song et al. identified that the TCA cycle and pentose phosphate pathway were potential mechanisms of the energy metabolism in human gastric cancer, which might be regulated through lysine succinylation in their core enzymes [43]. Furthermore, it is well known that thyroid hormone participates in energy regulation and metabolic processes, and the loss of thyroid hormone homeostasis is highly associated with various thyroid dysfunctions including hypothyroidism and hyperthyroidism [44, 45]. Thus, lysine succinylation may play a vital role in mitochondrial function and energy metabolism in the HFD-induced hypothyroxinemia rat model [38–41].

Fatty acids, as the major components of triglycerides, were found in increased contents in thyroid tissues of the HFD-induced rat model in previous studies. To further investigate the role of fatty acids in the mitochondrial function and to avoid cofounding factors *in vivo*, fatty acids were used to treat normal human thyroid epithelial cells, thus decreasing the succinylation level. Mitochondrial functions were then investigated *in vitro* in the present study. Palmitic acid, as the most common saturated fatty acid in animals, plants, and microorganisms, was applied. Nicotinamide (NAM), as an inhibitor of SIRT5, which has been reported to catalyze the removal of succinylation [46–48], was then treated to palmitic acid-stimulated cells to inhibit the desuccinylase activity of sirtuins. Mitochondrial OCR was then used to examine the mitochondrial function of normal human thyroid epithelial cells with altered succinylation levels. The results *in vitro* were in accordance with the results *in vivo*, further suggesting that fatty acids might directly influence energy metabolism and play an important role in the formation of hypothyroidism.

To the best of our knowledge, the present study is the first to investigate the succinylome in an HFD-induced hypothyroxinemia rat model. Succinylation level shows significant downregulation in many important proteins, mainly localized in mitochondria. We suppose that the significant succinylation downregulated mitochondrial ATP synthase complex, and mitochondrial IDH2 protein is more likely to accompany with the depressed tricarboxylic acid cycle activity because succinylation depends on intracellular succinyl-CoA levels, and succinyl-CoA can be generated from the TCA cycle, lipids, and amino acid metabolism, for succinylation can occur by a nonenzymatic chemical reaction.

## 5. Conclusions

This research reveals significant downregulated lysine succinylated proteins mainly localized in mitochondria and co-occur with the depressed mitochondria activity in the HFD-induced hypothyroxinemia rat model. These results expand our understanding of the underlying mechanism of hypothyroidism progression and provide new avenues of exploration with regard to potential treatment strategies for hypothyroidism.

## Data Availability

The data used to support the findings of this study are available from the corresponding author upon request.

## Supplementary Materials

Supplementary Materials Supplementary Table S1: fatty acids composition of diets. Supplementary Figure S1: Pearson's correlation coefficient and quality control of validation of MS/MS data. Supplementary Table S2: all annotated proteins identified and differentially expressed proteins in quantitative proteome of rat thyroid tissues; S2: HFD group and S1: CD group, each group of three biological repetitions. Supplementary Table S3: all annotated succinylated proteins and significantly differential lysine succinylation in quantitative succinylome of rat thyroid tissues. S2: HFD group and S1: CD group, each group of three biological repetitions.
