# Supplementary material for: Quantitative Analysis of the Proteome and the Succinylome in the Thyroid Tissue of High-Fat Diet-Induced Hypothyroxinemia in Rats
Source: Int J Endocrinol. 2020 Jul 23;2020:3240198. doi: 10.1155/2020/3240198 (PMC7396117; doi:10.1155/2020/3240198)
Supplement: Supplementary Materials — Supplementary Table S1: fatty acids composition of diets. Supplementary Figure S1: Pearson's correlation coefficient and quality control of validation of MS/MS data. Supplementary Table S2: all annotated proteins identified and differentially expressed proteins in quantitative proteome of rat thyroid tissues; S2: HFD group and S1: CD group, each group of three biological repetitions. Supplementary Table S3: all annotated succinylated proteins and significantly differential lysine succinylation in quantitative succinylome of rat thyroid tissues. S2: HFD group and S1: CD group, each group of three biological repetitions. [file 3240198.f1.zip › 3240198.f1/Supplementary Materials_Table S1, Figure S1.docx]

**Supplementary Table S1** Fatty acids composition of diets

| Fatty acids, g/Kg | CD | HFD |
| --- | --- | --- |
| C14:0 | 0.2 | 2 |
| C16:0 | 9.7 | 56.2 |
| C16:1n7 | 0.2 | 2.4 |
| C18:0 | 2.1 | 20.5 |
| C18:1n9c | 12.3 | 63.7 |
| C18:2n6c | 25.7 | 34.9 |
| C18:3n3 | 1.7 | 1.8 |
| C20:0 | 0.2 | 0.5 |
| C20:1 | 0.3 | 1.2 |
| Total saturated | 11.1 | 78.8 |
| Total mono-unsaturated | 12.2 | 67.8 |
| Total poly-unsaturated | 29.3 | 36.7 |
| Total | 52.6 | 183.3 |


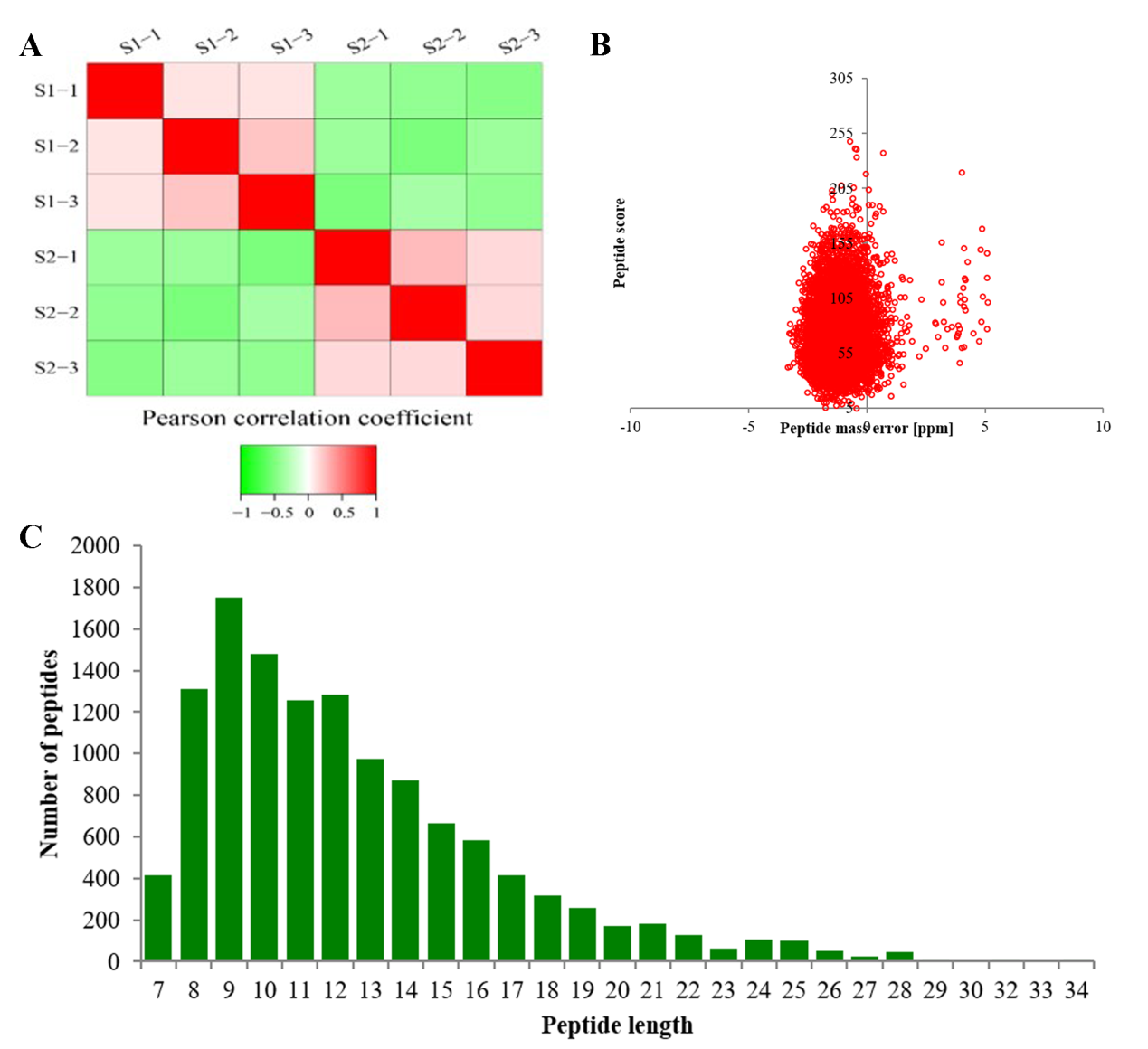
**Supplementary Figure S1** Pearson’s correlation coefficient and quality control of validation of MS/MS data. A. The heat map of Pearson's correlation coefficient between all samples. The S1 and S2 represented the replicates of Control group and HFD group, respectively. When the Pearson coefficient is closer to -1 that is a negative correlation, closer to 1 that is a positive correlation, the closer to 0 that is irrelevant. Red indicates that the correlation coefficient is 1, green indicates a correlation coefficient of -1, and white indicates a correlation coefficient of zero; B. Average peptide mass error; C. Length distribution of all identified peptides.
